# Damage Mechanisms and Mechanical Properties of High-Strength Multiphase Steels

**DOI:** 10.3390/ma11050761

**Published:** 2018-05-09

**Authors:** Sebastian Heibel, Thomas Dettinger, Winfried Nester, Till Clausmeyer, A. Erman Tekkaya

**Affiliations:** 1 Process Engineering and Materials, Mercedes-Benz Cars, Benzstraße, 71059 Sindelfingen, Germany; thomas.dettinger@daimler.com (T.D.); winfried.nester@daimler.com (W.N.); 2 Institute of Forming Technology and Lightweight Components, TU Dortmund University, Baroper Str. 303, 44227 Dortmund, Germany; Till.Clausmeyer@iul.tu-dortmund.de (T.C.); Erman.Tekkaya@iul.tu-dortmund.de (A.E.T.)

**Keywords:** damage, AHSS, UHSS, global formability, local formability, damage tolerance, edge-crack sensitivity, damage mechanics, fracture, bendability

## Abstract

The usage of high-strength steels for structural components and reinforcement parts is inevitable for modern car-body manufacture in reaching lightweight design as well as increasing passive safety. Depending on their microstructure these steels show differing damage mechanisms and various mechanical properties which cannot be classified comprehensively via classical uniaxial tensile testing. In this research, damage initiation, evolution and final material failure are characterized for commercially produced complex-phase (CP) and dual-phase (DP) steels in a strength range between 600 and 1000 MPa. Based on these investigations CP steels with their homogeneous microstructure are characterized as damage tolerant and hence less edge-crack sensitive than DP steels. As final fracture occurs after a combination of ductile damage evolution and local shear band localization in ferrite grains at a characteristic thickness strain, this strain measure is introduced as a new parameter for local formability. In terms of global formability DP steels display advantages because of their microstructural composition of soft ferrite matrix including hard martensite particles. Combining true uniform elongation as a measure for global formability with the true thickness strain at fracture for local formability the mechanical material response can be assessed on basis of uniaxial tensile testing incorporating all microstructural characteristics on a macroscopic scale. Based on these findings a new classification scheme for the recently developed high-strength multiphase steels with significantly better formability resulting of complex underlying microstructures is introduced. The scheme overcomes the steel designations using microstructural concepts, which provide no information about design and production properties.

## 1. Introduction

Body-in-white development is strongly driven by both targets reducing CO_2_ emissions and increasing crash safety. One way to reduce CO_2_ emissions via lightweight design is the reduction of component thickness. This is only possible with increasing materials strength. Therefore, a wide range of high-strength multiphase steels for various applications has been developed in the last decades. Their respective mechanical properties are directly related to the underlying thermo-mechanical processing and composition dependent microstructure. Characteristics of this microstructure are: phase distribution, phase morphology, phase hardness and hardness difference between phases, grain and particle size, texture and density of geometrically necessary dislocations [1]. These complex microstructures lead to a complex forming and fracture behaviour, which cannot be described only by the terms formability or rather ductility. Table 1 defines therefore the terms of global formability, local formability, damage tolerance, edge-crack sensitivity and fracture toughness for sheet materials.

Global formability as defined above is associated with a good in-plane forming behaviour, whereas local formability is accompanied with a good out-of-plane forming behaviour, for example, bending with high strain gradients in thickness direction and high local loading at the outer fibre, as well as good stretch flangeability. As damage is not failure [3], damage tolerance as defined here is the ability to undergo severe damage evolution during plastic flow, namely void nucleation, growth and coalescence, until rupture. Per definition, edge-crack sensitivity, with its tendency of a material to crack initiation due to further loading at a punched edge, is a special case of fracture toughness.

Multiphase steels are commonly classified according two systems. Many researchers divide them into first-, second- and third-generation AHSS (advanced high-strength steels), as displayed in Table 2 [4]. Another approach, displayed in Table 3, introduced by Euro Car Body Congress [5] is the assigning to the groups AHSS and UHSS (ultra high-strength steels).

The first generation of AHSS comprises classical dual-phase (DP), complex-phase (CP), martensitic (MS) and transformation induced plasticity (TRIP) grades. Higher strength levels in comparison to conventional mild steels are gained by substitution of the softer ferrite partially or completely by harder phases like bainite and martensite. In modern car body manufacturing among the first generation of AHSS DP steels are commonly used for structural components with mainly tensile loading like B-pillars or door beams. Typical forming technologies for this class of steel with its high global formability are deep drawing, stretching or hydroforming. The microstructure of classical DP steels is based on the distribution of hard martensitic islands in a soft ferritic matrix. The differing properties of the constituent phases as well as the crystallographic and chemical heterogeneity in ferritic grains lead to stress heterogeneity during forming which favours ductile damage evolution [1,6]. Tasan et al. [7] found that sharp deformation bands nucleate at ferrite grains and propagate in the softest route within the microstructure with angles of 45–50° to loading direction. This leads to very high strains in ferrite grains, which were determined by Ghadbeigi et al. using digital image correlation (DIC) and in-situ tensile testing inside a scanning electron microscope (SEM) for a DP1000 [8] as well as for a DP600 [9]. Ductile damage evolution is strongly dependent on the local microstructural morphology [1,10]. It is a dynamic process, where for example a crack of a martensite particle is followed by concentrated plastic deformation around that crack which triggers strain localization and local softening [11]. Besides martensite cracking voids nucleate in DP steels mainly by decohesion at ferrite-martensite interfaces. Ghadbeigi et al. [9] found martensite fracture, which is initiated at grain boundaries as main damage mechanism for a DP600. A DP800 investigated by Kadhkodapour et al. [12] displayed void nucleation by ferrite grain boundary decohesion. Ramazani et al. [13] classified martensite cracking as the main damage mechanism for a DP steel produced in a laboratory. Failure propagation along ferrite-martensite boundaries was found by Wang et al. [14] for a DP800 in contrast to a combination of martensite cracking and ferrite-martensite decohesion for a DP1000. In general strain heterogeneity and thus damage evolution lead to a reduced local formability which might lead to part fracture in the forming process in the form of edge-fracture, fracture with slight necking or fracture during bending on tight radii. Bainite can be introduced to the microstructure in order to improve the susceptibility to damage evolution [1,15]. Among the strengthening mechanisms grain refinement is the only one that can simultaneously improve the local formability of steels [1,16,17], whereas hereby the distribution of martensite becomes more critical and the ferrite-martensite morphology must be optimized [1,16]. Furthermore Hudgins et al. state that a reduction of hardness ratio is beneficial for improvement of classical DP-steels [18]. CP steels consist mainly of a fine-grained bainitic or tempered martensitic matrix with small amounts of other phases. With their lower hardness ratio CP grades exhibit on the one side a high local formability and on the other side due to the higher density of dislocation pile-ups because of the harder phases a poor global formability. Pathak et al. [19] showed for a CP800 the primary source for void nucleation at TiN (titanium nitride) particles and that secondary void nucleation takes place at martensite-bainite boundaries near the macroscopic failure strain. With its high yield strength and good local formability CP grades are used for structural parts with dominating compressive loads, for example, cross members or longitudinal members which are commonly produced by roll forming and bending operations. MS steels display an even higher yield strength than CP grades. They consist mainly of fine grained martensite and exhibit low necking strains, a poor global formability. The single phase microstructure leads to a homogenous strain distribution and thus to a good local formability with high local strains before fracture. TRIP steels were developed to achieve higher global formability in comparison to DP steels. They consist of a comparable microstructure with an added, certain amount of retained austenite, which is gained by a higher number of alloying elements. These alloying elements reduce the weldability and restrict automotive applications.

The same holds for the second generation of AHSS. The high manganese content of 15–20% enables a fully austenitic microstructure for the so-called twinning induced plasticity (TWIP) steels. The automotive applications are greatly limited by the as before mentioned poor weldability as well as by the tendency of delayed fractures.

Development of the third generation of AHSS focused on the improvement of global formability compared to 1. Gen. AHSS, without drawbacks of the 2. Gen. AHSS. Approaches to achieve this are quench and partitioning steels (Q&P) and TRIP aided bainitic ferrite steels (TBF) [20]. The latter consist mainly of bainitic ferrite and a certain amount of martensite. TBFs are produced by rapid cooling from an austenite microstructure into the bainitic regions followed by isothermal holding. According to [21] these steels are also named as DH steels, dual phase steels with higher global ductility/formability. The microstructure of Q&P steels consists of martensite, tempered martensite and bainite with retained austenite. One way to achieve this microstructure is by short cooling from austenite to partial martensite transformation followed by isothermal holding. According to the definition of DH steels, these steels can be also called CH, meaning complex-phase steels with higher global formability. Medium-manganese steels are under current development and therefore not yet listed in Table 2 and Table 3.

With improved microstructure of classical DP steels regarding damage effects as well as development of 3. Gen. AHSS the mechanical properties of modern AHSS are basically tailored for specific manufacturing processes and product applications. In this context, the whole set of mechanical properties related to the fracture behaviour (see Table 1) cannot be characterized only by the classical tensile test according to ISO 6892-1 [22].

The 3-point-bending test according to VDA 238-100 [23], originally developed for hemming assessment of aluminium alloys, has been established as state-of-the-art for characterization of bendability and crash ductility in automotive industry. Kurz et al. [24] showed a good correlation between determined bending angles and crash behaviour of press hardened steels (PHS). In recent research, Cheong et al. [25] improved the test set-up to determine local fracture strains for the plane-strain stress state via DIC besides the bending angle. Another way to achieve a plane-strain stress state is the usage of notched tensile specimens. For three different DP1000 grades Butcher et al. [26] detect, that local failure strains of a bending specimen, determined via DIC and a notched tensile specimen, determined via a combination of DIC and thickness measurement of the fracture surface, are almost similar. Strains of the notched tensile specimen are slightly lower, because the specimen exhibits a strain gradient in thickness direction, which is averaged by thickness measurement. Similar observations are found for press hardened steels [27]. To assess failure in bending operations in sheet metal forming simulation, Liewald et al. [28] introduced bending limit curves (BLC), which are falling linearly from left to right in the forming limit diagram. These lines are determined by performing DIC analysis on bending specimens according to VDA 238-100 [23] subjected to different levels of pre-straining. Another possibility for failure prediction in bending operations is using damage mechanics, for example the enhanced Lemaitre damage model, which was calibrated by Soyarslan et al. [29] on basis of notched tensile tests and used to predict fracture in bending of a DP600. 

Besides the bending test a further approach to predict the crash behaviour was introduced by Frometa et al. [30], who showed the correlation of fracture toughness formulated in terms of essential work of fracture with crash resistance in axial impact tests for several multiphase steels. In [31] Casellas et al. link fracture toughness of the same materials with results of hole expansion tests, which are typically conducted to access the edge-crack sensitivity. Comparable results have been published by Yoon et al. [32], who correlated fracture toughness of AHSS with stretch-flangeability.

The hole expansion test according to ISO 16630 [33] for characterizing these stretch-flangeability or rather edge-crack sensitivity itself displays several disadvantages and was therefore controversially discussed in recent research: In [34] the influences on the hole expansion ratio (HER) from the operator over the tool up to the evaluation procedure are presented. A European round robin test [35] revealed high deviations among several laboratories in testing of high strength steels because of the non-reproducible pre-damaged edge condition and the influence of several operators. Exemplarily, the HER showed a standard deviation of 14.6% with an average value of 58.8% for a martensitic steel [35]. Larour et al. [36] evaluated therefore alternative stretch flangeability testing methods.

Damage tolerance as defined in Table 1 is not characterized by a classical measure. Some damage and fracture models like Lemaitre [2] or the fracture forming limit line (FFL) approach [37] introduce a critical damage parameter $\;D_{crit}$, which can be interpreted as following: the higher the value of $\;D_{crit}$, the higher is the damage tolerance of the respective materials. The same holds for comparison of fracture strains predicted by advanced fracture criteria like the Modified-Mohr-Coulomb [38], Lou-Huh [39] or Hosford-Coulomb model [40]. The higher the level of the fracture line (in case of plane stress) or fracture surface for a material is, the more damage tolerant it is. Muenstermann et al. [41] introduced the ratio of fracture strain over strain at damage initiation computed with a modified Bai-Wierzbicki model in analysing a DP600 and DP1000 as measure for damage tolerance. According to this study the DP600 is much more damage tolerant than the DP1000, which can be explained by the higher amount of void nucleating microstructural constituents in DP1000.

The idea of global and local formability was used by Hance and Davenport [42] for presenting a new local and global formability map concept for application-specific material selection on examples of several AHSS of the strength class of 1000 MPa. In this proposal, the true uniform strain is used as measure for global formability. For local formability, the author suggested a so called true fracture strain, a strain measure for the reduction of area at fracture. Larour et al. [43] picked up this idea and used instead of the true fracture strain the Z-value according to ISO 6892-1 [22] and ASTM E6 [44] as measure for local formability. Using this measure, a good correlation to hole expansion ratios of punched as well as milled holes is obtained for a wide range of AHSS. 

The aim of this contribution is to introduce a physically motivated measure for local formability, which displays the possibility to qualitatively assess the bendability, fracture toughness, edge-crack sensitivity and damage tolerance, as these material characteristics are all influenced by the same microstructural parameters. Thus, the damage behaviour of 1. Gen. AHSS is characterized and a classification scheme using global and local formability is created. These results are transferred to several steels of 3. Gen. AHSS.

## 2. Theoretical Background on Formability Measures

**Figure 1:**
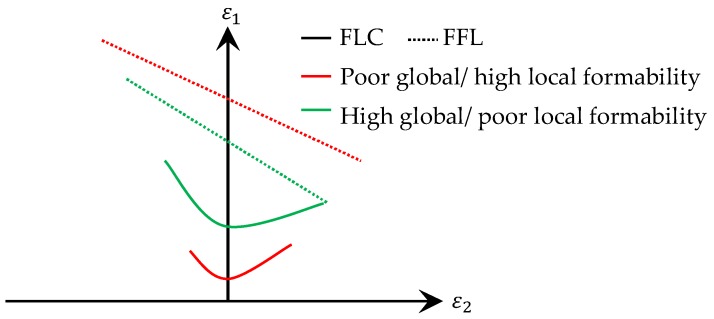
Schematic of FLC and FFL for materials displaying poor global/high local formability and vice versa.

Table 1 defines global formability as an ability of a material to undergo plastic deformation without formation of a localized neck. This corresponds well with the definition of the forming limit curve (FLC) [45,46], which is the classical tool for prediction of forming limits in sheet metal forming. This curve represents the onset of localized necking in principal strain space, commonly termed as forming limit diagram (FLD). In addition to the FLC Embury and Duncan [47] introduced fracture limits (e.g., FFL [37]) in the principal strain space, which fits into the definition of local formability with its ability of a material to undergo plastic deformation in a local area without fracture. Schematically these curves are displayed in Figure 1 for the two examples of poor global in combination with high local formability and vice versa.

### 2.1. Global Formability

Experimentally, the FLC is determined by several tests with Nakajima- or Marciniak-specimens between uniaxial and equi-biaxial tension. The high number of specimens in combination with a complex testing machine is the drawback of this approach as measure for global formability in material characterization.

The classical tensile test according to ISO 6892-1 [22] provides also information on the ability of a material to distribute strains uniformly in terms of the measure uniform strain. According to the Considère criterion [48], the true uniform strain $\varepsilon_{u}$ equals the hardening exponent n, which is commonly used to describe the formability of a certain material: the higher the n-value, the higher is the formability. As in [42,43], $\varepsilon_{u}$ in uniaxial tension test is used in this contribution as simplified measure for global formability, because of its lower complexity compared to determining FLCs. 

### 2.2. Local Formability

**Figure 2:**
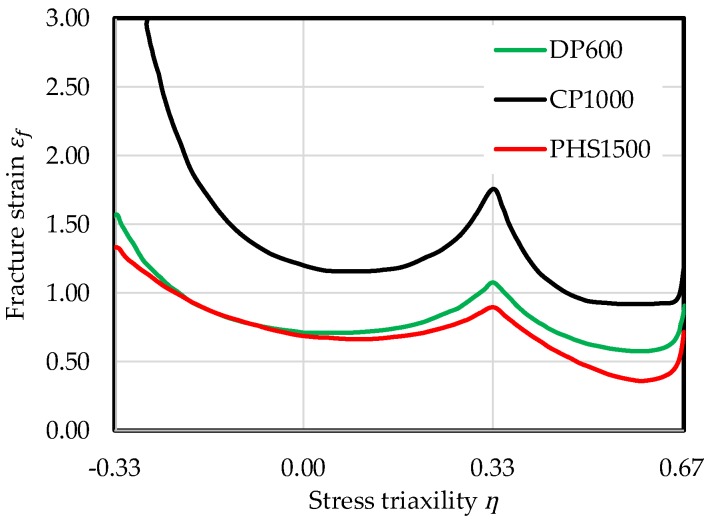
Fracture lines for DP600, CP1000 and PHS1500 [49].

AHSS display a damage induced reduced local formability which might lead to part fracture in the forming process in the form of edge-fracture or plane-strain failure, for example, fracture with slight necking or fracture on tight radii. One way to quantify the reduced formability is local measurement of fracture strains, which are used for example in damage models and fracture criteria. The resulting fracture surface or line, especially the level, could be easily used as measure for local formability, which is shown schematically for FFLs in Figure 1 and exemplarily for a DP600, a CP1000 and a press hardened steel with tensile strength of 1500 MPa (PHS1500) in fracture lines of the CrachFEM model in Figure 2 [49]. Especially in the stress state of plane-strain (stress triaxiality *ƞ* ≈ 0.58), the materials can be differed and classified well with the fracture strain as measure for local formability.

As for the FLC and global formability, the high number of specimens makes this approach costly in material characterization. Further possibilities, summarized in Table 4, are measures from damage mechanics and the measures for local formability introduced by Hance and Davenport [42] and Larour et al. [43] taking a tensile test into account. Hereby $r_{m}$ is the mean normal anisotropy, $\varepsilon_{3f}$ is the third-principal strain at fracture (true thickness strain at fracture), with

(1) $$\varepsilon_{3f}=\ln\left(t_{0}/t_{f}\right)$$

$t_{0}$ being the initial sheet thickness, $t_{f}$ the sheet thickness at fracture, *F_fracture_* the force at fracture elongation, *F_max_* the force at uniform elongation, $A_{0}$ the initial cross-sectional area and *A_f_* the cross-sectional area after fracture of tensile test according to ISO 6892-1 [22]. Detailed information on measuring of $A_{f}$ is given in [42,43]. 

All measures have in common, that they are based on fracture thickness. This holds even for $D_{crit}^{Lem}$, which incorporates the thickness information intrinsically via $F_{fracture}$ Martins et al. [37] state that the thickness reduction at fracture is independent of the loading history and therefore a material property, which was shown experimentally by Isik et al. [50] on an aluminium AA1050-H111 sheet by determining its FFL. The trend of falling from left to right in the principal strain space of the FFL (see Figure 1) is similar to the BLC [26], which is reasonable because of the link between the fracture strain of a notched tensile specimen and a bending specimen [26,27]. Both approaches, the FFL and BLC assess fracture under plane-strain conditions and differ only in their characterization procedure. Even the tensile specimen according to ISO 6892-1 [22] exhibits a non-proportional loading path of uniaxial tension during uniform elongation to plane-strain tension between the onset of localized necking and fracture. During localized necking the material flows from thickness in loading direction without a change in direction of the second principal strain. In combination with the definition of local formability in Table 1 this effect favours $\varepsilon_{3f}$ as measure for local formability in contrast to $A_{f}$, which takes also the width information into account. A further advantage of $\varepsilon_{3f}$ is, that the third-principal strain does not change its direction during loading. 

## 3. Materials and Methods

Firstly, the microstructural composition based on electron backscatter diffraction analysis (EBSD) and micro-hardness measurements, the engineering stress-strain curves as well as the bending angles of the commercially produced study materials are presented. Hereon the methods for examining the damage and failure mechanisms of the core investigated materials DP600, DP800, CP800, DP1000 and CP1000 are described in detail. Afterwards the tests for assessing global and local formability are specified. The materials with higher global formability and/or higher strength, namely DH600, DH800, DH1000, CH1000, DH1200, CP1200 and CH1200, are investigated with a reduced program. Table A1 in Appendix A summarizes the investigated materials with short names, designation according to VDA 239-100 [51] and initial sheet thickness.

### 3.1. Materials

The chemical composition of the investigated materials lays in the range as specified in VDA 239-100 [51]. The microstructural composition is determined as basis of the investigations in this contribution. Therefore, EBSD measurements have been conducted at the Central Facility for Electron Microscopy at RWTH Aachen. Table 5 summarizes the results. Bainite and tempered martensite cannot be distinguished via EBSD analysis. The underlying phase-illustrations of the microstructure are presented in Table A2. The grades with higher global formability display a high amount of retained austenite, which is responsible for the “TRIP”-effect during loading [21]. The amounts lay in a range from 2.6% (CH1200) up to 10.7% (DH1200). The microstructure of the DP600 corresponds well to a “classical” DP steels microstructure with martensite islands in a ferrite matrix (grain size 4.04 µm) including martensite lines in the centre plane of the sheet. The DP800 and DP1000 exhibit higher amounts of bainite, 30.1% respectively 59.5%. Because of the higher amount of bainite, as previously noted in [1,15] and in combination with the small grain size, both grades are already improved regarding their susceptibility to damage evolution. The “balanced” fraction of phases makes it in this case more suitable to term these DP steels more general as multiphase steels.

The CP grades with a tensile strength ≥1000 MPa show an amount of bainite or tempered martensite of more than 90%. In contrast, the CP800 differs just slightly from the DP800. Besides DP600, the DH600 shows martensite lines in the sheets centre plane. Qualitatively, the microstructure of DP1000 and DH1200 are most heterogeneous regarding phase distribution and martensite morphology.

In addition to phase fractions and grain size determined by EBSD analysis micro-hardness measurements according to Martens as indicator for local formability are used to characterize the microstructure. A cylindrical indenter after Vickers with an opening angle of 136° (ISO 6507-1 [52]) is hereby indented with a force of 1 mN into the specimen. The Vickers Hardness HV is calculated via the indention depth according to ISO 14577-1 [53]. For every material three specimens (longitudinal cut) with three measurement rows (each with 100 measuring points) are conducted. One row lays in the specimen centre and the other rows 0.2 mm above and below the central row. The quantity specified as “HV” presented in Table 5 represents the mean value of these 900 measuring points. The hardness difference ∆HV, as quantitative measure for heterogeneity, is the difference between 20% of the highest and 20% of the lowest of these 900 measured values. The CP grades with their homogenous bainitic/tempered martensitic microstructure display the lowest hardness differences with values <200 HV, whereas DP and DH grades show values >300 HV. Exceptions are the DH600 with 208 HV and the DH1000 with 253 HV. 

**Figure 3:**
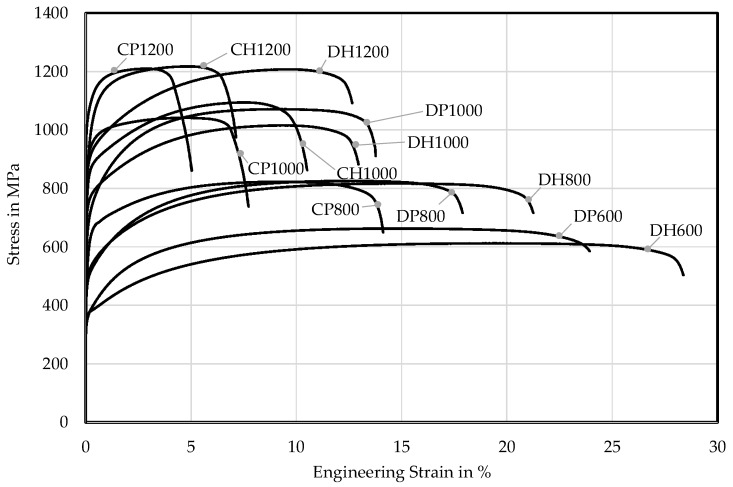
Engineering stress-strain curves (LD) according to SEP 1240 [54].

The macroscopic mechanical properties are tested by the uniaxial tensile test according to SEP 1240 [54] with specimen geometry defined in EN ISO 6892-1 [22] as well as by performing the three-point bending test according to VDA 238-100 [23]. The resulting engineering stress-strain curves longitudinal to the sheets rolling direction (LD) are illustrated in Figure 3. The mechanical properties are detailed in Table A3 and Table A4. There is a correlation between the microstructural composition and the mechanical properties. DP and DH steels exhibit higher uniform elongation (A_G_) and total elongation (A_80mm_) than the CP and CH grades in the comparable strength classes. Because of their higher hardness difference and higher amount of ferrite DP and DH steels show a lower initial density of dislocation pile-ups. The stress-drop before fracture resulting from their lower hardness differences and more homogenous microstructure displays an indicator for the higher local formability of the CP grades. Due to rolling in the production process the longitudinal specimens show higher uniform and fracture strains than the transversal ones, which can be stated vice versa for the stresses. This effect can be also seen in the bending angles α according to VDA 238-100 (Table 6), which are lower if the bending line coincides with the longitudinal direction. The bending angle varies mostly between strength classes, in-between a certain strength class, the bending angles are quite similar. Differences between DP/DH and CP/CH can hardly be seen. In Table A5 micrographs of the bending specimens are displayed. Only the DH800, CP800, DP1000, DH1000, DH1200 and CH1200 show significant cracks at the failure criterion of a 30 N load drop, where the bending angle is measured according to VDA 238-100. For the CP1200 and CH1000 one can detect ductile incisions, a failure step in bending before developing of a crack. DP600, DH600, DP800 and CP1000 show no failure at all. 

### 3.2. Methods

**Figure 4:**
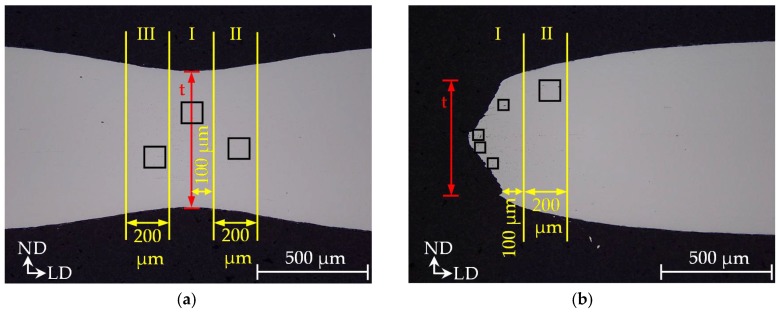
Measurement of porosities: necked specimen (**a**); fractured specimen (**b**).

**Figure A1:**
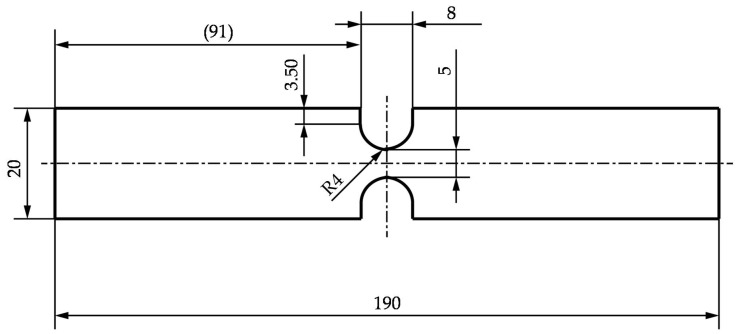
Geometry of the notched specimen.

Fracture in sheet metal forming, for example, fracture at tight radii or fracture with underlying non-linear loading paths and prior localized necking takes mostly place in the stress-state of plane-strain. This holds also for fracture under crash loading, for example, compressive loading of a longitudinal member or tensile loading in a B-pillar. This stress-state can be reached in laboratory with a bending (e.g., VDA 238-100 [23]), a grooved tensile or notched tensile specimen. The latter is used for damage and failure characterization in this contribution. The specimen’s geometry is detailed in Figure A1 in Appendix B. The notched tensile tests are executed longitudinal and transversal to rolling direction with the investigated core materials DP600, DP800, CP800, DP1000 and CP1000. The longitudinal specimens are used afterwards for investigations of the main damage and failure mechanisms. After conducting the tests till fracture, the tests are stopped therefore at the point reaching maximum force as well as at 50%, 75%, 85%, 90% and 95% of fracture displacement. Micrographs of longitudinal sections in the specimen’s centre are prepared to quantitatively and qualitatively assess damage evolution via light microscopy (LM) and SEM. The preparation of the micrographs is detailed in Table A6 and Table A7 with details of the influence of grinding and polishing steps in Table A8. The measurement of porosities to quantify the damage evolution is illustrated in Figure 4. In case of a necked specimen the micrograph is divided into three parts based on the thinnest thickness t. The true thickness strain $\varepsilon_{3}$ is measured via t in part I. In every part squares of 100 µm × 100 µm are created at the position of highest number of voids. The porosities are then measured in the three parts via grey-scale analysis. Fractured specimens are divided into two parts. In part I four squares of 50 µm × 50 µm are generated. For measurements of porosities in part II a 100 µm × 100 µm square is created equally to a necked specimen. 

To explore the underlying damage mechanisms, nital etched micrographs of the fractured specimens are investigated via SEM. Furthermore, an EBSD analysis is conducted for a at 95% of fracture displacement stopped DP600 specimen. 

**Figure A2:**
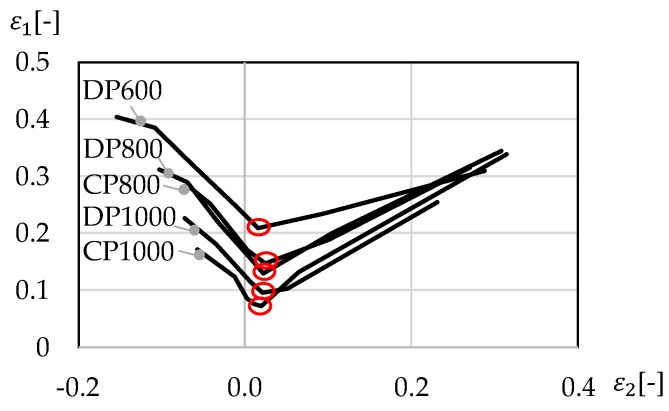
Forming limit diagram.

**Figure A3:**
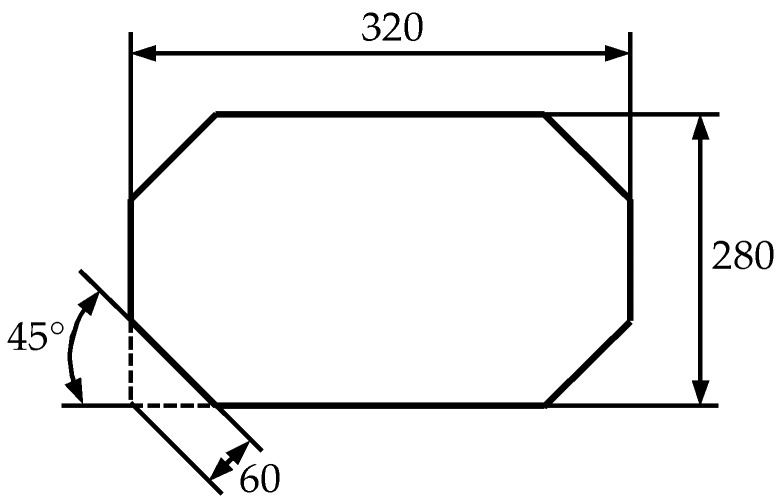
Initial blank geometry for cross-die samples.

The results of damage and failure analysis are used then as basis for the classification scheme in terms of mechanical properties in Section 5: global and local formability. Hereby measures transversal to loading direction are used to represent the “worst” case with smallest bearable strains. As stated in Section 2.1 the true uniform strain $\varepsilon_{u}$ is used in this research as measure for global formability. To confirm and validate this measure, it is correlated with the hardening exponent (n-value), the minimum of FLC ($\varepsilon_{1min}$) and the maximum drawing depth of cross-die samples without failure. The FLCs of the five investigated core materials are displayed in Figure A2. The curves are obtained by tests with Nakajima specimens according to ISO 12004-2 [55]. For the forming trials of the cross-die samples the same initial blank size (Figure A3: long side equals rolling direction) is used for every material. The blanks are lubricated with a forming oil (Oest Platinol). The results are summarized in Table A9. Every material exhibits ductile fracture with prior localized necking and typical dimples on the fracture surface as can be seen in Table A10. 

**Figure 5:**
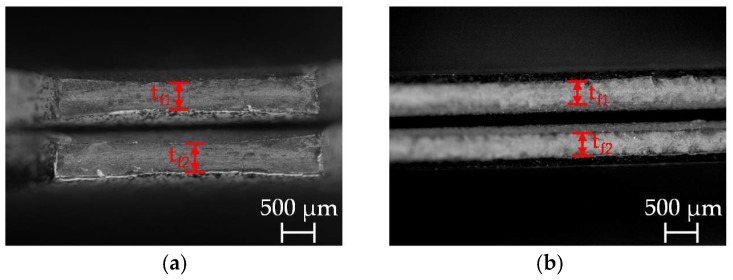
Measurement of fracture thickness: notched specimen (**a**); A_80mm_ specimen (**b**).

The true thickness strain at fracture $\varepsilon_{3f}$ of the uniaxial tensile test (A_80mm_) is used here as measure for local formability as discussed in Section 2.2. For confirmation and validation of this measure it is correlated with the true thickness strain at fracture of the notched tensile specimen for the investigated core materials and for all study materials with the hole expansion ratio according to ISO 16630 [32]. The measurement of fracture thickness via light microscopy to derive $\varepsilon_{3f}$ according to Formula (1) is illustrated in Figure 5. Both parts of the broken specimen are measured simultaneously at the thinnest position and a mean value for the particular specimen is generated. The results of the hole expansion tests are shown in Table A11. 

**Figure 6:**
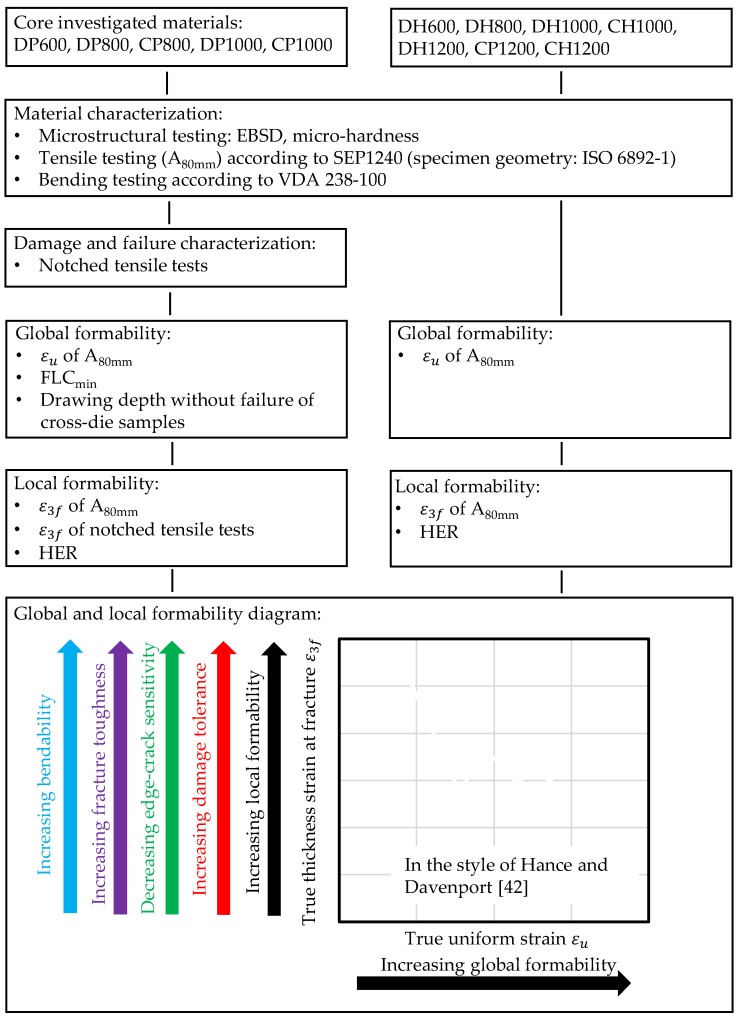
Methodology of investigations.

Finally, a global-local formability diagram in the style of [42] is created. Based on this diagram the mechanical material properties global formability, local formability, damage tolerance, edge-crack sensitivity, fracture toughness and bendability can be estimated and assessed. Figure 6 summarizes this methodology. 

## 4. Damage and Failure Characterization

**Figure 7:**
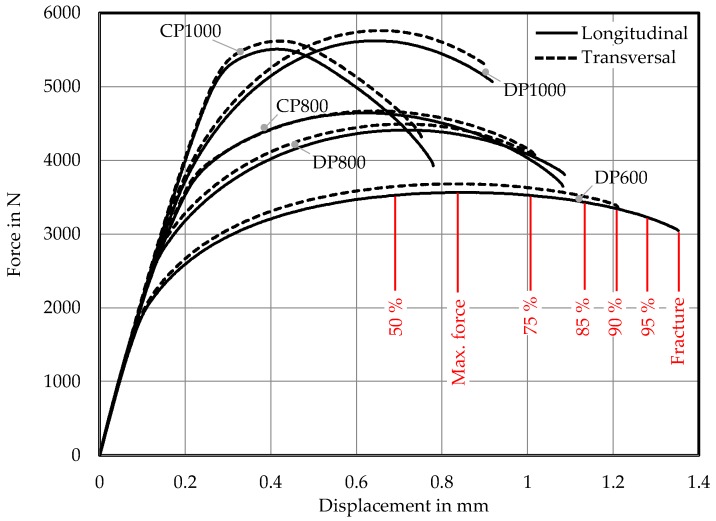
Force–displacement diagram of notched tensile tests; Red lines indicate discrete levels of stopped tests for the example of DP600.

Damage and failure characterization is mainly based on the notched tensile specimen (Figure A1). The force-displacement curves are displayed in Figure 7. As outlined in Section 3.2 the positions of stopped tests marked exemplarily for DP600. Tests were stopped at these positions to obtain micrographs of each grade. Longitudinal and transversal to rolling direction tested specimens show similar characteristics like in the uniaxial tensile test (Figure 3, Table A3 and Table A4). Firstly, damage in terms of porosity is quantified. Then the damage and failure mechanisms are evaluated and the results are discussed in context of references from literature.

### 4.1. Quantification of Damage Evolution

**Figure 8:**
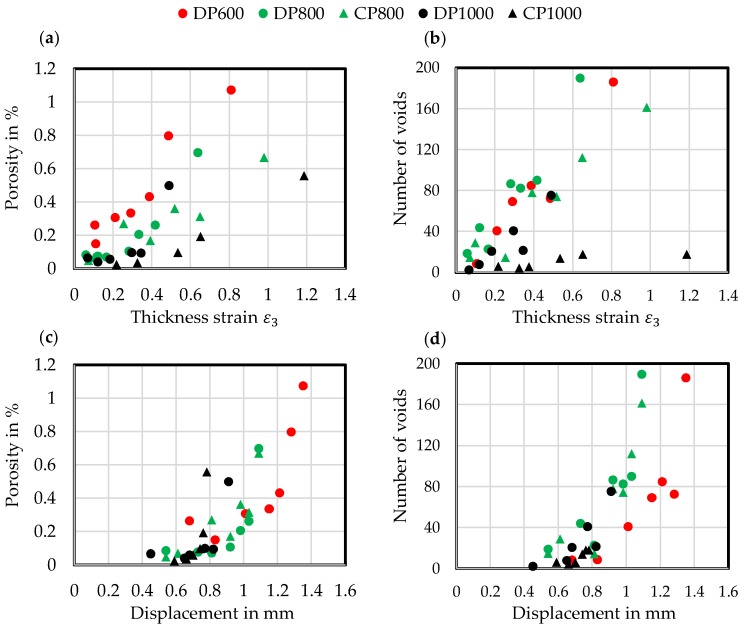
Notched tensile test: (**a**) Porosity–Thickness strain diagram; (**b**) Number of voids–Thickness strain diagram; (**c**) Porosity–Displacement diagram; (**d**) Number of voids–Displacement diagram.

Figure 8 summarizes the results in porosity—thickness strain (a), number of voids—thickness strain (b), porosity—displacement (c) and amount of voids-displacement (d) diagrams. Micrographs are detailed in Appendix C Table A12, Table A13, Table A14, Table A15 and Table A16. The displayed points are mean values out of three specimens analysed as described in Section 3.2. In general, nearly linear relationships between porosity and number of voids with thickness strain can be observed as well as nearly exponential relationships between porosity and number of voids with displacement, which is quite obvious, because of the well-known exponential increase of thickness strain during localized necking. Thus, it can be stated that damage evolution for these steels is strongly driven by an increase in strain during localized necking. DP steels show higher porosities and higher amounts of voids while exhibiting lower thickness strains than the CP grades, because of DP steels’ more heterogeneous microstructure with higher hardness differences between the phases, which lead to larger incompatibilities during loading. 

The DP600 displays the highest porosities and highest number of voids up to fracture resulting from the early void nucleation and from the high amount of nucleation sites due to finely dispersed martensite particles in the ferrite matrix with highest grain size of the investigated core materials. In Table A12 one can see large voids in the specimen’s centre, which nucleated early in the martensite lines. In contrast, the DP800 shows a quite similar number of voids but lower porosity values because of smaller in size voids due to the smaller ferrite grain size. For the CP800 the number of voids and porosity values are quite similar to the DP800 but the 15% higher amount of bainite, which leads to a more homogenous microstructure, shifts the endurable strains to higher levels and consequently reduces the local strain and stress gradients between the phases. The DP1000 displays lower porosities and a lower number of voids. The relationship between porosity and thickness strain deviates here the strongest from a linear one. The rate of porosity increase increases strongly after thickness strains of 0.3. The higher amount of martensite in this steels microstructure leads to sudden fracture due to damage at lower local strains, which will be detailed in Section 4.2. The CP1000 shows the lowest number of voids with quite high porosities. The homogeneous bainitic/tempered martensitic microstructure with low hardness difference makes this steel damage tolerant and capable of accommodating high local strains. Large voids results from nucleation at hard inclusions at low strains and growth driven by very high local strains during localized necking.

### 4.2. Damage and Failure Mechanisms

SEM micrographs (Table 7) are used to analyse the damage and failure mechanisms in detail. The DP steels exhibit void nucleation mainly due to martensite particle fracture with few nucleation sites between martensite and ferrite resulting from differing phase properties, which lead itself to strain heterogeneity. Damage evolution, namely void growth and coalescence, takes place along grain boundaries and martensite rows. In CP steels voids nucleate mainly between martensite and the matrix as well as due to inclusion-matrix decohesion. The DP600 shows depending on its local microstructural morphology both nucleation between martensite and ferrite and due to martensite particle fracture. Damage evolution takes place along grain boundaries and along martensite lines. The evolution along grain boundaries and not through ferrite grains can be detected clearly in micrographs of EBSD analysis in Table A17. Because of the high amount of martensite the main void nucleation mechanism of the DP1000 is martensite particle fracture. Shear bands localize during loading in ferrite grains in the microstructure [7]. This shear band localization leads to an increase in local strains and strain gradients, which itself leads to highly increased damage initiation and evolution and thus final fracture. The DP1000 with its high amount of martensite shows because of this phenomenon sudden fracture at relatively low strains, when the limit strains against martensite particle fracture are reached along the shear bands. Final material separation (ductile fracture) is the result of ductile damage evolution in combination with local shear banding as can be seen at the 45° fracture pattern for the materials in Table 7. These fracture patterns can be also seen in the bending specimens (Table A5), where the cracks also occur in direction of local shear bands (45° to ND). The cross-die samples (Table A10) show also ductile fracture as stated before. The damage and failure process is similar again. The SEM micrographs of the fracture surfaces show smaller voids with increasing strength for the DP grades and vice versa for the CP grades, which correlates well with the observations in the notched tensile specimen. The 45° fracture pattern of the micrographs displays again the existence of shear bands which lead together with ductile damage mechanisms to final fracture. Additionally, the thickness at fracture is quite similar to these of the notched tensile specimens.

**Figure A4:**
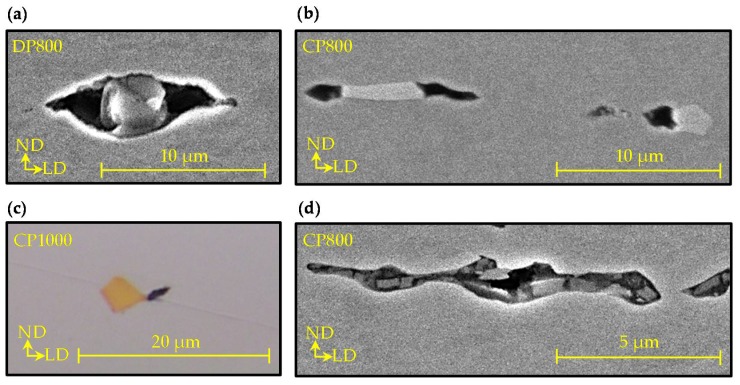
Examples for damage nucleation at inclusions: (**a**) aluminium oxide; (**b**) niobium carbide; (**c**) titanium carbide; (**d**) manganese sulfide.

Inclusions play a minor role in this damage and failure process. Nevertheless, examples are shown in Figure A4. Voids nucleate at the hard inclusions (aluminium oxide, niobium carbide and titanium carbide) early in the loading process, which leads to big voids. These inclusions are present in a too low amount to influence failure during the forming process or in crash loading. Same holds for the soft manganese sulphides.

### 4.3. Discussion

The investigated materials show all common damage mechanisms. These damage mechanisms and whole damage evolution are strongly dependent on the local microstructural morphology as stated before by [1,10]. For this reason, the damage mechanisms observed in [8,9,10,11,12,13,14] for DP steels between 600 MPa and 1000 MPa and the damage mechanisms detected in this study differ from each other. The tensile strength and other mechanical properties defined in norms like the VDA 239-100 [51] can be reached with different material concepts and thus different microstructural compositions, so that the damage mechanisms exemplarily for a DP600 must not be similar for two different products. Nevertheless, the experimental observations for DP600 and DP1000 lay in good accordance with the modified Bai-Wierzbicki model based analysis of Muenstermann et al. [38]. In their study, the DP600 is much more damage tolerant than the DP1000 which fits well with the results presented here. The macroscopic event of ductile fracture is on the whole induced by a combination of shear banding, which was initially found by Tasan et al. [7] and ductile damage evolution, which is strongly driven by localized necking (holds not for equi-biaxial tension where localized necking is suppressed), at a characteristic, macroscopic measurable fracture strain, for example, thickness strain. From a fracture mechanical point of view, the fracture of high-strength multiphase sheet steels can be classified as a combination of ductile normal fracture (Mode I) and through thickness shear fracture (Mode III).

## 5. Characterization and Classification in Terms of Mechanical Properties—Global and Local Formability

Multiphase steels with a heterogeneous microstructure exhibit a damage induced reduced formability, measurable in fracture strains, while high-strength steels with a homogeneous microstructure display a poor global formability and a good damage tolerance. To build a classification scheme for these steels, first of all the measures for global and local formability are confirmed for the investigated study materials as outlined in Section 3.2. The resulting global and local formability diagram is then completed and explained with the rest of the materials.

### 5.1. Global Formability

**Figure 9:**
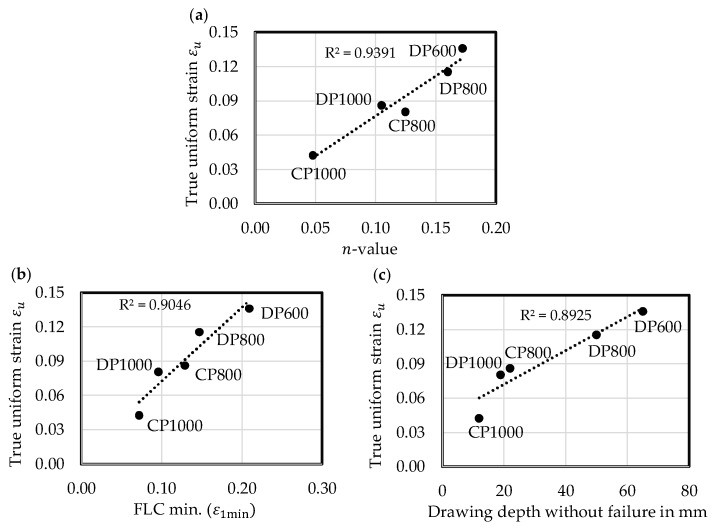
True uniform strain $\varepsilon_{u}$ as measure for global formability: (**a**) Correlation with n-value; (**b**) Correlation with forming limit curve (FLC) min.; (**c**) Correlation with drawing depth without failure of cross-die samples.

The true uniform strain as measure for global formability is confirmed and validated by correlation with n-value, with the minimum of the FLC ($\varepsilon_{1min}$) and the maximum drawing depth without failure of the cross-die samples. There are positive correlations for all measures as illustrated in Figure 9. The true uniform strain equals not exactly the n-value, which could be expected due to the complex hardening behaviour of multiphase steels.

Although the FLC represents the beginning of localized necking and $\varepsilon_{1min}$ represents the plane-strain stress state, the simplification with the A_80mm_ specimen works well and the correlation with true uniform strain in the uniaxial stress state is positive. The cross-die samples show independently of the location of ductile fracture and although the underlying loading paths are non-linear and not similar for the materials localized necking in combination with the in Section 4.2 described ductile damage and failure process. The positive correlation of true uniform strain with the drawing depth favours the true uniform strain gained by conducting tensile tests with an A_80mm_ specimen as parameter for global formability. It displays a qualitative boundary to the beginning of the ductile damage and failure process, which is covered by local formability.

### 5.2. Local Formability

**Figure 10:**
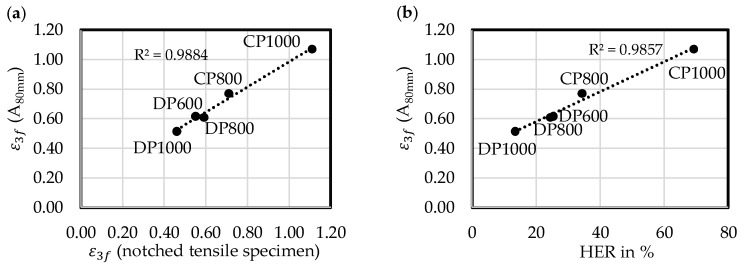
True thickness strain at fracture $\varepsilon_{3f}$ (A_80mm_) as measure for global formability: (**a**) Correlation with $\varepsilon_{3f}$ (notched tensile specimen); (**b**) Correlation with hole expansion ratio (HER).

The macroscopic event of ductile fracture is initiated at a characteristic fracture strain. Among these fracture strains the true thickness strain at fracture $\varepsilon_{3f}$ is advantageous as stated in Section 2.2. The diagram in Figure 10a correlates $\varepsilon_{3f}$ (A_80mm_) with $\varepsilon_{3f}$ measured in the notched tensile specimens. Expect for CP1000, the values derived with the A_80mm_ specimen are slightly higher. The good correlation shows that $\varepsilon_{3f}$ (A_80mm_) is a suitable measure for local formability. This holds also for bendability because the stress-states at fracture are in both cases plane-strain, which is shown experimentally by Butcher et al. [26]. Furthermore, it is expected by damage models [2,29] and fracture criteria [28,37,38,39,40,41] that the fracture strains of a plane-strain tensile specimen and the fracture strains at the outer fibre of a bending specimen are nearly the same respectively only slightly lower in case of the tensile specimen. 

**Figure A5:**
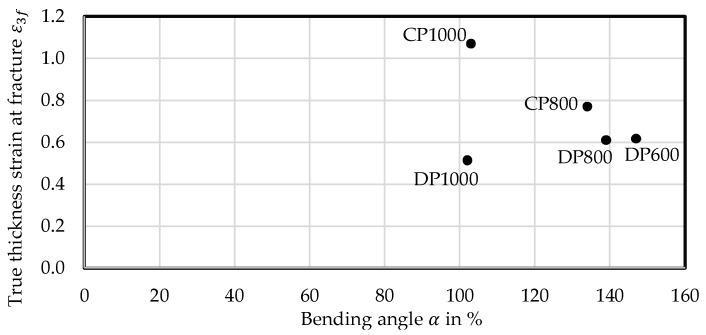
Local formability: No correlation between true thickness strain at fracture $\varepsilon_{3f}$ and bending angle α according to VDA 238-100 [23].

Figure A5 in Appendix D shows no correlation between the true thickness strain at fracture and the bending angle α according to VDA 238-100 [23]. The failure criterion of 30 N load drop seems to be unsuitable for the studied materials with a sheet thickness of 1.00 mm respectively 1.20 mm. This becomes obvious in Table A5, where five materials show no fracture as well as from the detailed analysis of DP1000 and CP1000 in Table A18. The materials display bending angles of 103° and 104° respectively when reaching the failure criterion. However, in CP1000 cracks with a length first comparable to DP1000 are reached for bending angles >150°. Moreover, the bending angle according to VDA 238-100 [23] is more a macroscopic measure than a local one. Though, fracture under bending is a local process on the outer fibre. Therefore, the measurement of local fracture strains on the bending line, as conducted by [25] would be a more characteristic measure for bendability instead of the bending angle α. Another reason favouring the local bending strain or the related radius as an objective measure for the material-specific performance in bending is the fact that different materials exhibit a substantially different bending line. The curvature of the sheet is homogeneous for DP1000 in the vicinity of the punch compared to CP1000 with an almost horizontal tangent (see Table A5 and Table A18). Nevertheless, the bending angle might correlate well with the crash behaviour under compressive loads for thicker materials which is not evaluated in this contribution.

The diagram in Figure 10b shows a good correlation between $\varepsilon_{3f}$ derived by the uniaxial tensile test and the hole expansion ratio, which makes $\varepsilon_{3f}$ also suitable to estimate qualitatively the edge-crack sensitivity of high-strength multiphase steels. Because of the link found by Frometa et al. [30] and Yoon et al. [33] between HER and fracture toughness, $\varepsilon_{3f}$ is capable to assess qualitatively the fracture toughness at room temperature.

### 5.3. Characterization and Classification Scheme: Global and Local Formability Diagram

**Figure 11:**
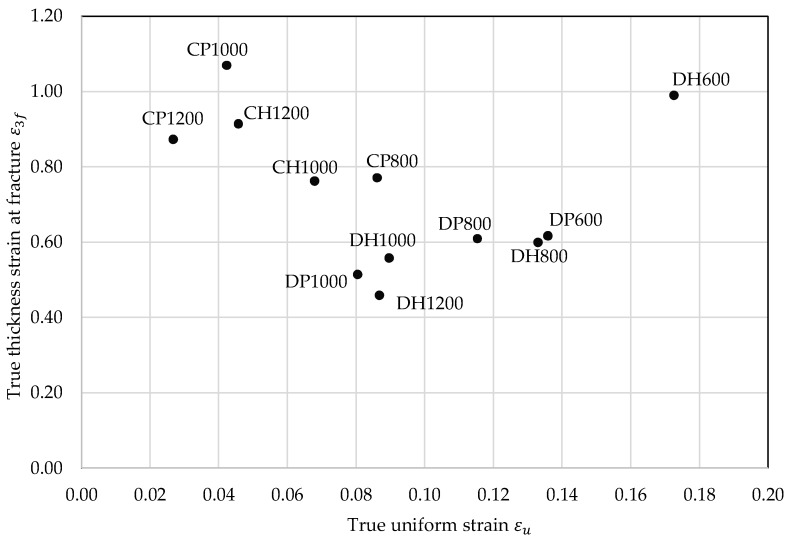
Global and local formability diagram: true thickness strain at fracture–true uniform strain.

**Figure A6:**
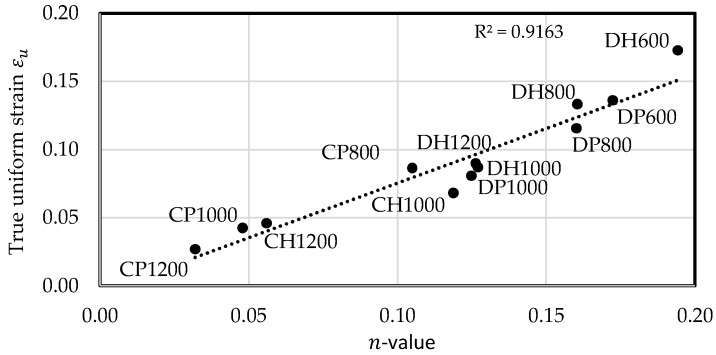
Global formability: Correlation between true uniform strain and n-value.

**Figure A7:**
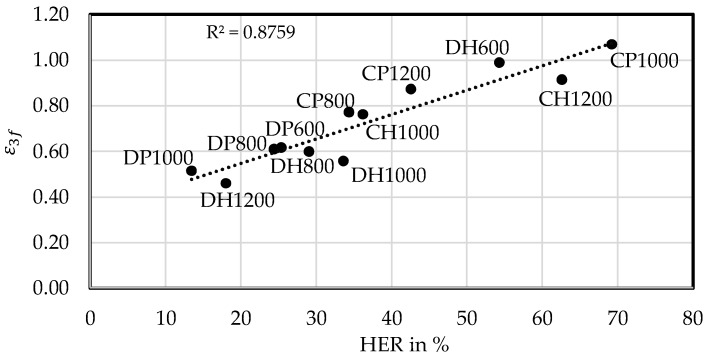
Local formability: Correlation between true thickness strain at fracture $\varepsilon_{3f}$ and hole expansion ratio HER.

Based on both derived measures for global and local formability a diagram (see Figure 11) is generated incorporating all investigated materials. Diagrams for correlation of $\varepsilon_{u}$ with the n-value and $\varepsilon_{3f}$ with HER for all investigated materials are illustrated in Figure A6 and Figure A7. DP600 and DH600 show the highest global formability, which is even higher for the DH600 because of the retained austenite fraction of 5% in the steel’s microstructure. In addition, local formability and thus damage tolerance is very high for the DH600 because of the low difference in hardness between the phases and thus the more homogenous stress and strain distribution. Noticeable in the SEM micrographs of both materials, displayed in Table A19, is the occurrence of bigger voids for the DH grade, which is similar for all DH- and CH-grades and might result from a damage mechanism related to the retained austenite. The global formability of the DH800 is on nearly the same level as the DP600′s, because of the “TRIP”-effect [21] during loading related to the retained austenite. The local formability remains on nearly the same level compared to the DP800’s. The CP800 displays a higher local formability as expected and lower global formability in comparison to the DP800, because of its more homogenous microstructure. The local formability is obviously reduced for the DP1000 due to its lower damage tolerance in comparison to DP800 resulting from the higher amount of martensite in its heterogeneous microstructure.

Both local and global formability appear slightly improved for the DH1000. Despite the higher tensile strength, the DH1200 shows a higher global formability than the DP1000 because of its high amount of retained austenite. The even more heterogeneous microstructure with a high amount of martensite of 16.1% leads to a poor local formability for the DH1200. Expectedly the CP1000 exhibits the highest local formability with its homogenous bainitic/tempered martensitic microstructure and low difference in hardness. Because of its higher tensile strength, the CP1200 shows a lower global formability than the CP1000 and with a higher difference in hardness despite the smaller grain size an even lower local formability. The local formability is slightly improved for the CH1200 because of its lower difference in hardness. In comparison to this steel the local formability of CH1000 is reduced because of its more heterogeneous microstructural composition, which let it in combination with 6.4% retained austenite reach a value for global formability on nearly the same level as the DP1000.

### 5.4. Discussion

**Figure 12:**
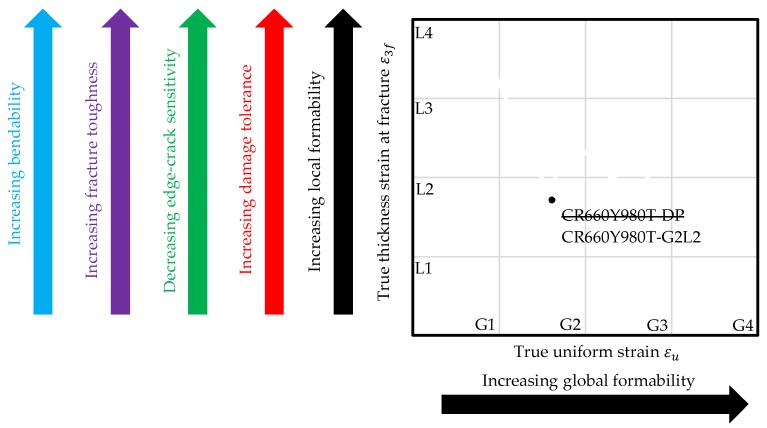
Designation on basis of global and local formability.

The global and local formability diagram based on uniaxial tensile testing enables the assessment of the mechanical material response regarding the failure and fracture behaviour according to Table 1 (global and local formability, damage tolerance, edge-crack sensitivity, fracture toughness and bendability) incorporating all microstructural characteristics on a macroscopic scale. Under consideration that a designation based upon phase composition is not suitable anymore, the diagram favours a new designation of multiphase steels on the basis of the mechanical properties. One possible proposal is illustrated in Figure 12. The diagram is divided in style of a chessboard with delimited fields. Instead of naming the DP1000 CR660Y980T-DP according to VDA 239-100 [51] the steel could be named based on the coordinates of the field as CR660Y980T-G2L2 (global 2/local 2). The designation based on mechanical properties enables the correct material selection in the early design stage with respect to specific forming processes and crash loading cases: Whether a good in-plane forming behaviour is needed (global type), bending operations have to be conducted or edge-cracks must be prevented (local type) as well as with respect to crash loading cases with dominating tensile (global type) or compressive loads (local type). Furthermore, it supports lightweight design by the possibility of replacing for example a DP600 with a DH800 and thus enables downsizing. Another advantage of this classification scheme is the possibility to differ materials with the same tensile strength regarding their damage tolerance, local formability and the other in Table 1 listed, related properties, for example, dual-phase steels with a tensile strength of 600 MPa but different underlying microstructures. In addition, the diagram reveals one drawback of classical failure assessment in sheet metal forming simulation with the FLC, which covers only global formability. The potential of steels with a high local and poor global formability, for example, CP1000 in bending dominated forming operations, is not completely used.

The conclusions drawn by $\varepsilon_{3f}$ as measure for local formability might be improved, if the fraction of $\varepsilon_{u}$ on $\varepsilon_{3f}$ is excluded:

(2) $$\varepsilon_{3f}^{*}=\varepsilon_{3f}-\varepsilon_{u}/\left(1+r\right)$$

**Figure A8:**
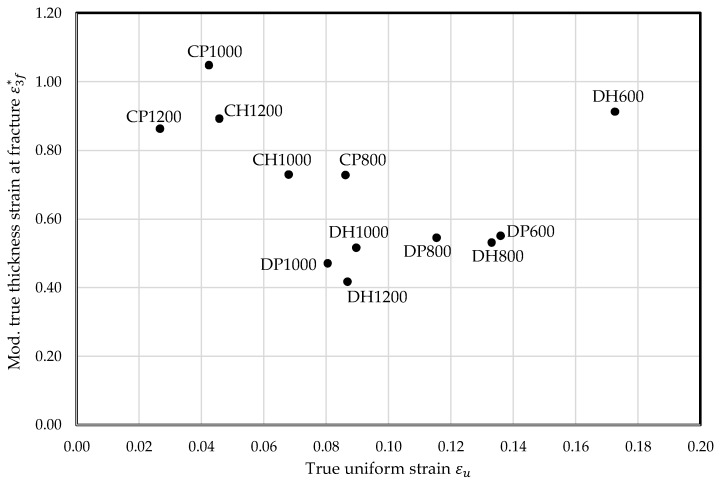
Modified global and local formability diagram: modified true thickness strain at fracture—true uniform strain.

The effect on the study materials is marginal as can be seen in Figure A8. The usage of $\varepsilon_{3f}^{*}$ could become important for high-strength steels showing for example TWIP effects because of their high uniform elongation. Though the global and local formability diagram can be built on various measures for local formability, $\varepsilon_{3f}$ and $\varepsilon_{3f}^{*}$ are advantageous in comparison to the true fracture strain (TFS) [39] or reduction of area (Z-value) [40]. The thickness strains do not take the fracture width into account, which is only influenced by global formability, because after localized necking the material in a tensile specimen flows from thickness into tensile direction. Thus, the statement regarding local formability becomes more distinctive. 

## 6. Conclusions

Based on damage and failure analysis a new characterization and classification scheme for high-strength multiphase steels has been introduced. Ductile damage mechanisms are strongly dependent on the local microstructural morphology. Nevertheless, ductile fracture takes place at a characteristic, macroscopic measurable thickness strain at fracture as result of a combination of these ductile damage mechanisms and local shear band localization. The true thickness strain at fracture is suitable as measure for local formability, which allows also the estimation of damage tolerance, edge-crack sensitivity, fracture toughness and bendability. Together with the true uniform strain as measure for global formability a characterization and classification scheme can be created which incorporates all microstructural characteristics via two parameters on a macroscopic scale. The resulting diagram can be used also as basis for a new designation of high-strength multiphase steels, which displays advantageous regarding information about design and production properties.

Future work will focus on damage mechanisms of DH- and CH-grades as well as on an analysis of dependency of the ductile fracture behaviour and thus of the characterization and classification scheme based on global and local formability on sheet thickness.

## Figures and Tables

**Table 1:** Definition of terms regarding the mechanical properties related to fracture behaviour of high-strength multiphase steels.

Term	Definition
Global formability	Ability of a material to undergo plastic deformation without formation of a localized neck respectively to distribute strains uniformly
Local formability	Ability of a material to undergo plastic deformation in a local area without fracture
Damage	Decrease of the load-bearing capacity of a material due to the appearance and evolution of voids [2]
Damage tolerance	Ability of a material to undergo severe damage evolution until rupture
Edge-crack sensitivity	Tendency of a material to crack initiation due to further loading at a punched edge
Fracture toughness	Ability of a material to withstand the growth of existing cracks
Bendability	Ability of a material to undergo bending operations without crack initiation along the bending line

**Table 2:** 1., 2. and 3. Gen. AHSS.

Group	Grades
1. Gen. AHSS	DP, CP, MS, TRIP
2. Gen. AHSS	TWIP
3. Gen. AHSS	TBF (DH), Q&P (CH)

**Table 3:** AHSS and UHSS.

Group	Grades
AHSS	DP, TRIP, TWIP, TBF (DH)
UHSS	CP, MS, Q&P (CH)

**Table 4:** Measures for local formability.

Measure	Reference	Definition
Critical damage parameter ($D_{crit}^{FFL}$)	Martins et al. [37]	$D_{crit}^{FFL}=\frac{1+r_{m}}{3}\varepsilon_{3f}$
Critical damage parameter ($D_{crit}^{Lem}$)	Lemaitre [2]	$D_{crit}^{Lem}=1-\frac{F_{fracture}}{F_{max}}$
True fracture strain (*TFS*)	Hance and Davenport [42]	$TFS=\ln\frac{A_{0}}{A_{f}}$
Reduction of area (Z)	Larour et al. [43]	$Z=\frac{A_{0}-A_{f}}{A_{0}}$

**Table 5:** Microstructural composition.

Material	Ferrite in %	Bainite ^1^ in %	Martensite in %	Ret. Austenite in %	Ferrite Grain Size in μm	HV	∆HV
DP600	83.2	10.5	4.4	0.8	4.04	343	350
DH600	79.3	11.6	3.1	5.0	3.45	341	208
DP800	61.4	30.1	5.1	2.9	1.67	413	331
DH800	59.6	28.1	4.3	6.6	1.73	440	380
CP800	51.4	44.9	1.3	2.0	1.37	388	219
DP1000	27.3	59.5	9.5	2.3	1.26	454	326
DH1000	20.6	67.9	2.6	7.8	1.36	490	253
CP1000	4.3	93.5	0.9	0.3	0.98	466	118
CH1000	6.3	84.4	1.5	6.4	0.97	512	195
DH1200	7.9	62.2	16.1	10.7	1.46	526	309
CP1200	3.3	95.1	0.1	0.3	0.76	574	195
CH1200	4.5	91.3	0.3	2.6	0.84	543	174

^1^ Bainite or tempered martensite, both phases cannot be distinguished via EBSD measurements.

**Table 6:** Bending angles according to VDA 238-100 (failure criterion: 30 N load drop) [23].

α	DP600	DH600	DP800	DH800	CP800	DP1000	DH1000	CP1000	CH1000	DH1200	CP1200	CH1200
LD	147°	148°	139°	117°	134°	103°	104°	102°	108°	81°	84°	90°
TD	151°	153°	145°	133°	144°	102°	113°	104°	116°	95°	92°	98°

**Table 7:** Damage mechanisms (scanning electron microscopy (SEM) micrographs): (1) Void nucleation between martensite and matrix; (2) Void nucleation due to martensite particle fracture; (3) Void nucleation due to inclusion-matrix decohesion; (4) Damage evolution along grain boundaries; (5) Damage evolution along martensite rows.

DP600	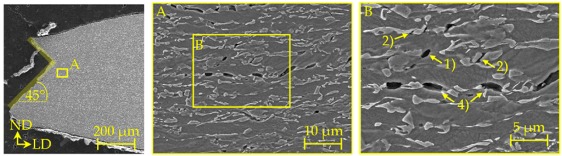
DP800	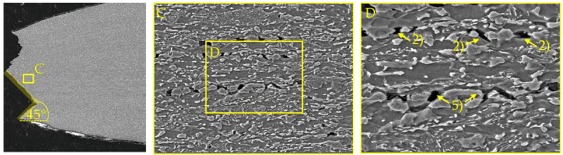
CP800	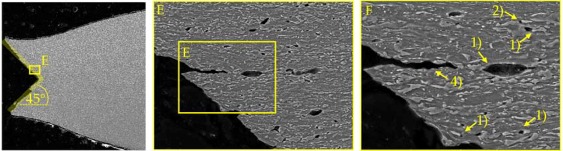
DP1000	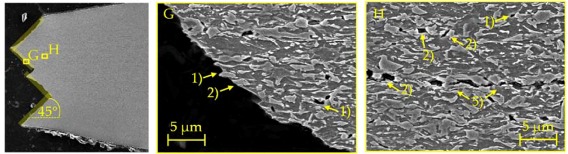
CP1000	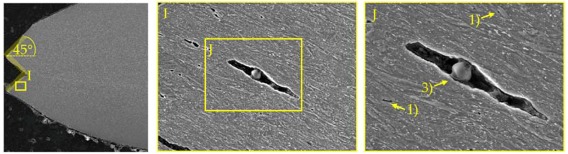

## Appendix A

**Table A1:** Investigated materials.

Short Name	Designation after VDA 239-100	Thickness in mm
DP600	CR330Y590T-DP-GI50/50-U	1.00
DH600	CR330Y590T-DH-GI50/50-U	1.00
DP800	CR440Y780T-DP-GI50/50-U	1.00
DH800	CR440Y780T-DH-GI50/50-U	1.00
CP800	CR570Y780T-CP-GI50/50-U	1.00
DP1000	CR660Y980T-DP-GI50/50-U^1^	1.00
DH1000	CR700Y980T-DH-GI50/50-U	1.00
CP1000	CR780Y980T-CP-GI70/70-U	1.00
CH1000	CR780Y980T-CH-EG53/53-U^1^	1.20
DH1200	CR850Y1180T-DH-EG53/53-U^1^	1.00
CP1200	CR900Y1180T-CP-EG53/53-U	1.20
CH1200	CR900Y1180T-CH-UC-U^1^	1.00

^1^ Not specified yet, designation according to VDA 239-100 [51].

**Table A2:** Microstructure (EBSD measurements).

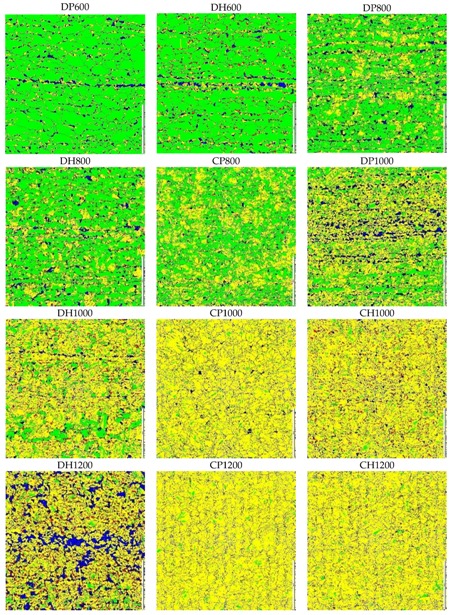

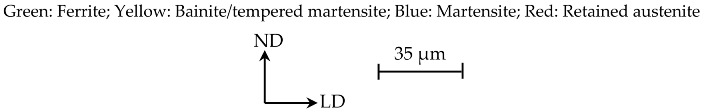

**Table A3:** Mechanical properties longitudinal (to rolling) direction (LD).

Material	R_p0.2_ in MPa	R_m_ in MPa	R_p0.2_/R_m_	A_G_ in %	A_80_ in %	n_2-A_G__	r_2-A_G__
DP600	377	658	0.573	14.76	24.03	0.17	0.89
DH600	376	615	0.616	19.75	28.78	0.20	0.99
DP800	510	824	0.619	12.63	18.12	0.17	0.71
DH800	520	808	0.643	14.74	21.36	0.17	0.79
CP800	643	825	0.779	9.44	15.46	0.11	0.81
DP1000	708	1060	0.668	9.00	14.01	0.13	0.70
DH1000	737	976	0.755	9.57	13.13	0.14	0.95
CP1000	925	1029	0.897	4.60	7.36	0.05	0.96
CH1000	838	1079	0.777	7.53	10.48	0.13	1.02
DH1200	896	1200	0.747	9.64	12.68	0.14	0.96
CP1200	1076	1199	0.898	2.89	5.07	0.03	1.51
CH1200	956	1200	0.797	4.84	7.10	0.06	1.16

**Table A4:** Mechanical properties transversal (to rolling) direction (TD).

Material	R_p0.2_ in MPa	R_m_ in MPa	R_p0.2_/R_m_	A_G_ in %	A_80_ in %	n_2-A_G__	r_2-A_G__
DP600	371	664	0.558	14.56	24.04	0.17	1.08
DH600	375	614	0.611	18.84	28.78	0.18	1.24
DP800	530	833	0.636	12.23	17.72	0.16	0.81
DH800	805	805	0.634	14.23	20.64	0.16	0.97
CP800	691	857	0.807	8.90	13.79	0.11	1.01
DP1000	714	1084	0.659	8.38	11.88	0.12	0.86
DH1000	764	1000	0.764	9.38	13.53	0.13	1.18
CP1000	932	1026	0.908	4.33	6.31	0.05	0.93
CH1000	832	1057	0.787	7.03	9.93	0.12	1.09
DH1200	899	1220	0.737	9.07	12.03	0.13	1.09
CP1200	1072	1207	0.888	2.71	4.69	0.03	1.77
CH1200	951	1184	0.803	4.68	7.03	0.06	1.15

**Table A5:** Micrographs of bending specimens (LD).

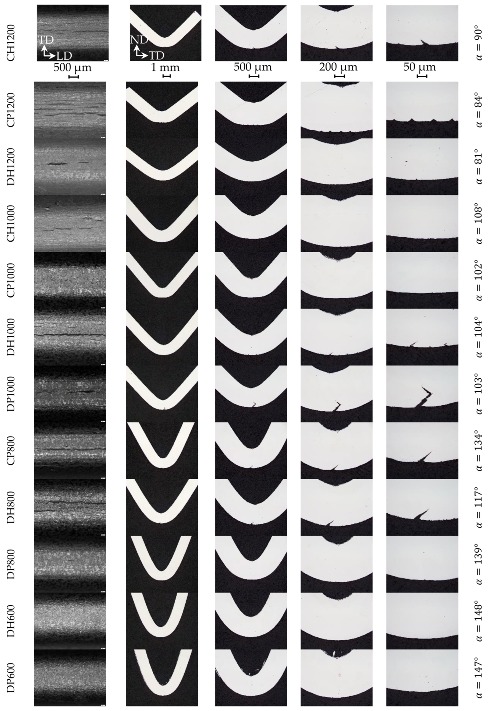

## Appendix B

**Table A6:** Grinding steps.

Step	Details
1	210 N, 300 U/min, 55 s, 180-grit
2	210 N, 300 U/min, 55 s, 320-grit
3	210 N, 300 U/min, 55 s, 600-grit
4	210 N, 300 U/min, 55 s, 800-grit
5	210 N, 300 U/min, 55 s, 1200-grit
6	210 N, 300 U/min, 55 s, 2500-grit

**Table A7:** Polishing steps.

Step	Details
1	15 N, 150 U/min, 6 min, 9 µm grain size
2	15 N, 150 U/min, 6 min, 6 µm grain size
3	15 N, 150 U/min, 6 min, 3 µm grain size
4	15 N, 150 U/min, 4 min, 1 µm grain size
5	15 N, 150 U/min, 3 min, 0.25 µm grain size diamond suspension
6	15 N, 150 U/min, 3 min, 0.05 µm grain size oxidic polishing suspension

**Table A8:** Influence of grinding and polishing steps on the example of DP600.

2500-Grit	6 µm Grain Size	3 µm Grain Size	1 µm Grain Size	0.05 µm Grain Size

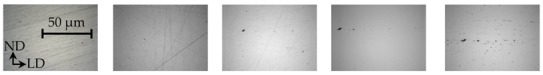

**Table A9:** Drawing depths without failure (necking or fracture) of cross-die samples.

Setting	DP600	DP800	CP800	DP1000	CP1000
Drawing depth	65 mm	50 mm	22 mm	19 mm	12 mm
Blank holder force	1200 kN	1000 kN	200 kN	200 kN	100 kN

**Table A10:** Ductile fracture of cross-die samples.

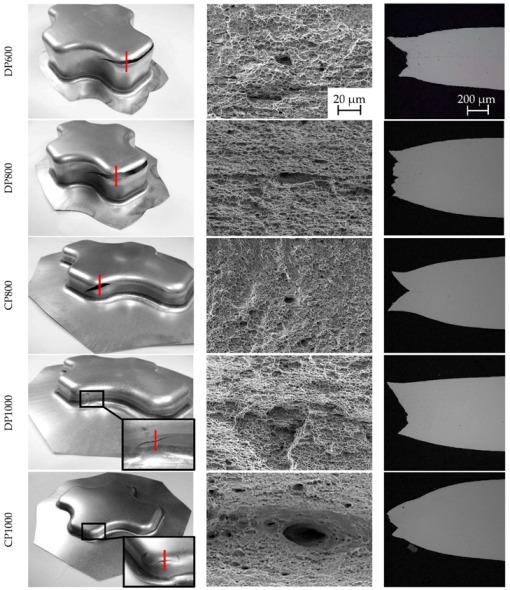

**Table A11:** Hole expansion ratios (HER) λ according to EN ISO 16630 [32] (*n* = 30).

HER	DP600	DH600	DP800	DH800	CP800	DP1000	DH1000	CP1000	CH1000	DH1200	CP1200	CH1200
λ	25.37	54.28	24.40	29.05	34.36	13.46	33.58	69.23	36.18	18.00	42.56	62.62
s	2.68	4.17	2.38	2.66	4.47	2.07	4.26	8.19	6.05	1.62	12.13	8.72

## Appendix C

**Table A12:** Damage evolution of DP600.

Fmax	
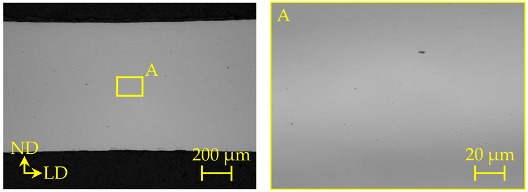
95%	
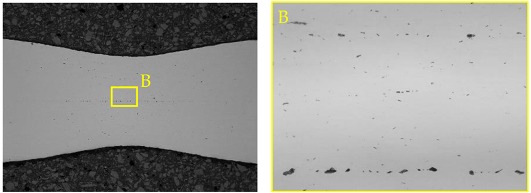
Fracture	
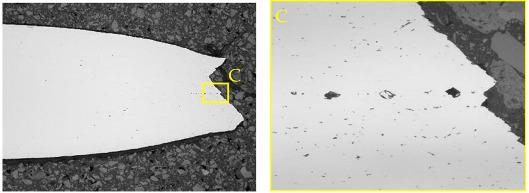

**Table A13:** Damage evolution of DP800.

Fmax	
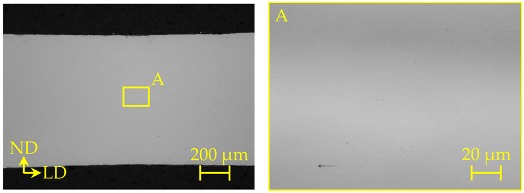
95%	
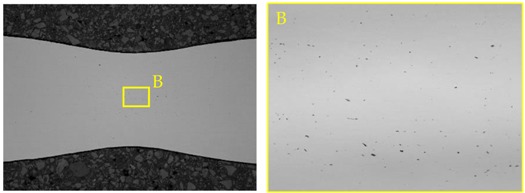
Fracture	
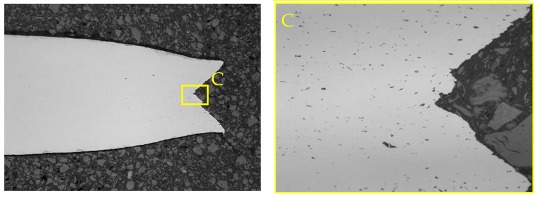

**Table A14:** Damage evolution of CP800.

Fmax	
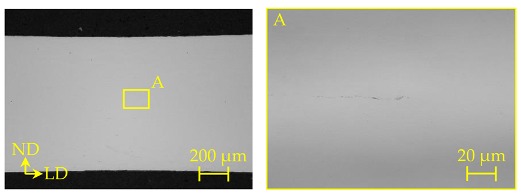
95%	
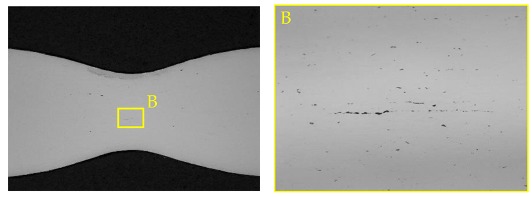
Fracture	
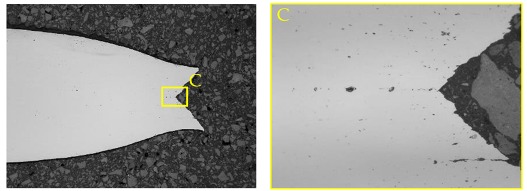

**Table A15:** Damage evolution of DP1000.

Fmax	
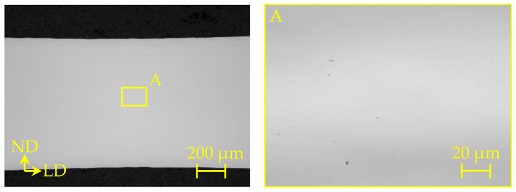
95%	
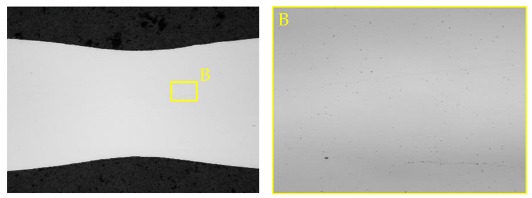
Fracture	
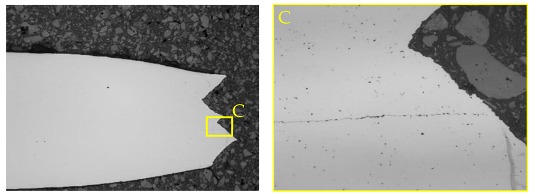

**Table A16:** Damage evolution of CP1000.

Fmax	
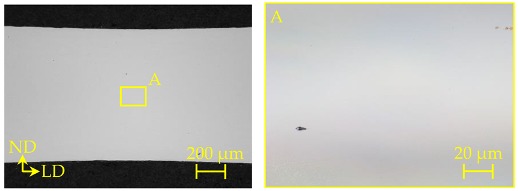
95%	
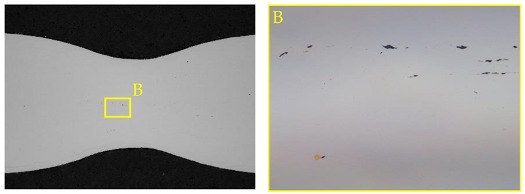
Fracture	
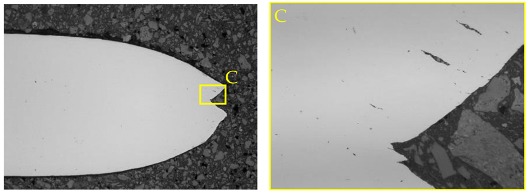

**Table A17:** Damage evolution along grain boundaries DP600.

Initial State	95%

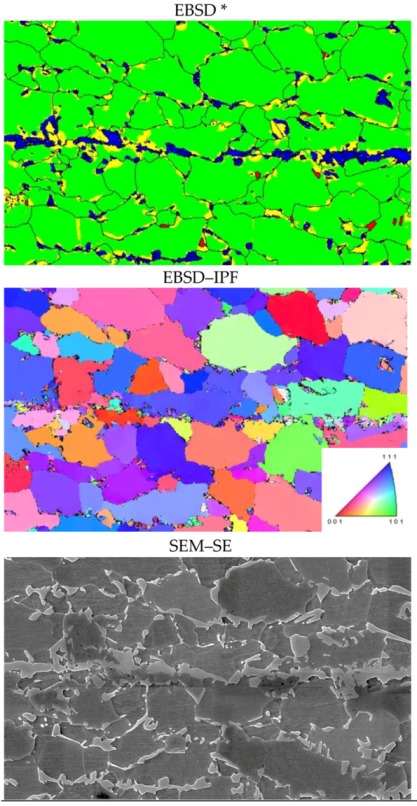
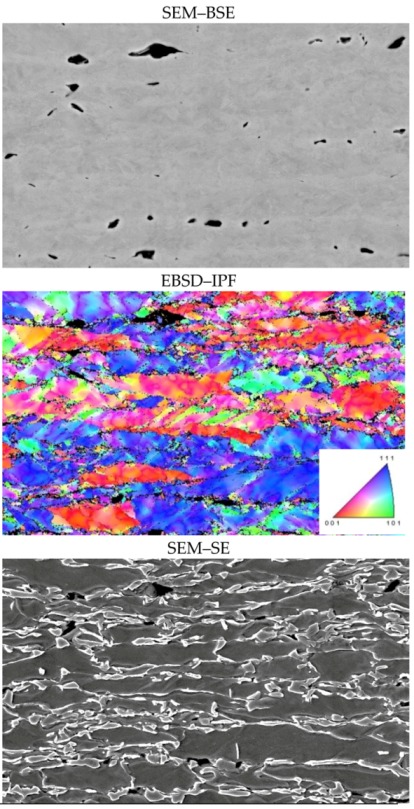
	
* Green: Ferrite; Yellow: Bainite/tempered martensite; Blue: Martensite; Red: Retained austenite	
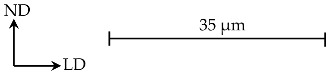

## Appendix D

**Table A18:** Micrographs of bending specimens (LD: Load drop, PD: Punch displacement).

DP1000			CP1000			CP1000		
α	LD	PD	α	LD	PD	α	LD	PD
103°	30 N	10.4 mm	104 °	30 N	10.5 mm	156°	-	15.5 mm

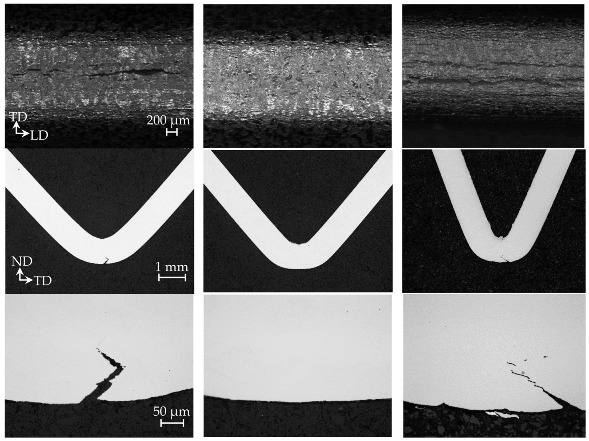

**Table A19:** Fracture surfaces A_80mm_-specimens.
